# The Remission Therapy Inquiry-Based Research Model (RIRM): a conceptual framework for mechanistic hypothesis generation

**DOI:** 10.3389/fsysb.2026.1835804

**Published:** 2026-07-08

**Authors:** Pichit Suvanprakorn, Krit Pongpirul

**Affiliations:** 1 Thai Red Cross Society, Bangkok, Thailand; 2 Center of Excellence in Preventive and Integrative Medicine (CE-PIM), Faculty of Medicine, Chulalongkorn University, Bangkok, Thailand; 3 Department of Infection Biology & Microbiomes, Faculty of Health and Life Sciences, University of Liverpool, Liverpool, United Kingdom

**Keywords:** cognitive analytics, conceptual discovery, grounded theory, inquiry-based learning, mechanistic reasoning, remission, reproducibility of reasoning, systems medicine

## Abstract

**Background:**

Modern biomedical research excels at validating hypotheses but often struggles with conceptual discovery. The emphasis on statistical significance has fostered a culture of measurement without mechanism—abundant evidence, yet limited progress toward durable restoration of health. The Remission Therapy Inquiry-Based Research Model (RIRM) was developed to address this gap by reframing how scientists reason about the mechanisms through which biological systems achieve functional coherence and recovery.

**Concept:**

In RIRM, True Remission is used not as a biological endpoint but as a conceptual lens for mechanistic inquiry. The framework guides researchers to identify the mechanistic origins of clinical phenomena—whether cellular, molecular, systemic, adaptive, or emergent through interacting biological systems—and to reason explicitly about how functional coherence may be restored. This process is organized through twelve self-inquiry questions and a route-based algorithmic roadmap that make early-stage scientific reasoning explicit, traceable, and open to examination.

**Perspective:**

RIRM bridges qualitative and quantitative epistemologies by integrating the interpretive depth of grounded theory with the structural discipline of systems medicine. Rather than replacing empirical validation, it strengthens the conceptual foundations that precede experimentation. By shifting emphasis from the reproducibility of outcomes alone toward the transparency, coherence, and reproducibility of reasoning, RIRM positions mechanistic inquiry itself as a legitimate domain of scientific rigor in biomedical research. As a conceptual framework, it offers a structured approach for organizing, teaching, evaluating, and refining mechanistic hypotheses across multiple levels of biological interpretation.

## 1 Introduction

Medical research has long equated progress with statistical success. The achievement of a *p*-value below 0.05 often overshadows the deeper purpose of therapy: sustained restoration of health. In many clinical domains, outcomes are defined by transient symptom control or incremental biomarker shifts, while the underlying drivers of dysfunction remain unaddressed. This has created a paradox in modern medicine—abundant evidence, yet limited enduring cures (Ioannidis, 2005; Goodman et al., 2016).

The notion of remission occupies an uneasy space within this landscape. In oncology, it denotes the absence of detectable disease; in autoimmune and metabolic conditions, it often describes a temporary reprieve. Yet across fields, remission rarely implies a return to biological normalcy. It is a descriptive rather than mechanistic term—an observation rather than an explanation of how restoration to health occurs. Biological examples such as Antarctic icefishes, which maintain organismal transport despite the evolutionary loss of hemoglobin through compensatory cardiovascular adaptations, illustrate that restoration of function need not always correspond to restoration of canonical molecular structures (Sidell and O’Brien, 2006). Restoration to health, therefore, may not always correspond to molecular or structural normalization but must instead be understood in terms of systemic functional coherence. Functional coherence refers to the integrated and coordinated operation of biological components and processes sufficient to maintain or restore effective system-level function, regardless of whether this is achieved through normalization, compensation, adaptation, or reorganization.

To engage this broader view, we introduce True Remission not as a biological endpoint but as a conceptual lens for mechanistic inquiry. Within the Remission Therapy Inquiry-Based Research Model (RIRM)—a systems-oriented conceptual framework developed in this study—True Remission is conceptualized as the restoration of coherent function through normalization, compensation, adaptation, or reorganization of biological processes. The central challenge in contemporary biomedical research is not merely to demonstrate that an intervention “works,” but to understand the mechanisms through which functional recovery is achieved across diverse clinical contexts (Popper, 1959; Kuhn, 1962). Addressing this challenge requires moving beyond population-level comparisons toward structured, mechanism-oriented reasoning. Unlike experimental methods or analytical tools, RIRM is intended to structure and make explicit the reasoning processes that precede empirical validation.

RIRM was developed to scaffold this process. It is a structured framework that guides researchers in identifying the mechanistic origin of a clinical problem—whether cellular, molecular, systemic, adaptive, or emergent—and in conceptualizing how functional coherence might be restored. Rather than prescribing hypotheses, RIRM organizes inquiry through twelve structured questions and an algorithmic reasoning roadmap. In doing so, it transforms self-inquiry into a documented and potentially reproducible process for organizing mechanistic reasoning.

In this framework, the term “mechanistic” refers broadly to explanatory processes linking clinical phenomena to underlying biological, physiological, systemic, adaptive, or interacting system dynamics. Mechanistic reasoning within RIRM is therefore not restricted to molecular pathways alone, but includes multi-level causal organization across cells, tissues, organs, regulatory systems, and adaptive biological responses.

**FIGURE 1:**
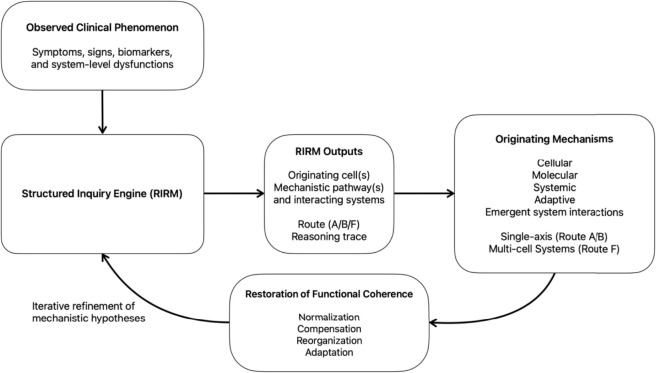
Overview of the Remission Therapy Inquiry-Based Research Model (RIRM).

Figure 1 provides an overview of the RIRM, illustrating how observed clinical phenomena—including symptoms, signs, biomarkers, and system-level dysfunctions—initiate a structured inquiry process that generates explicit mechanistic outputs, including candidate originating cells, mechanistic pathways, interacting systems, and reasoning traces. These outputs support classification of underlying mechanisms and inform potential pathways toward restoration of functional coherence. By explicitly separating observation, inquiry, and mechanistic understanding, RIRM clarifies how diverse biological, systemic, adaptive, and emergent processes may underlie similar clinical presentations. The framework allows mechanistic explanations to involve single or interacting pathways across multiple biological scales rather than assuming isolated linear causality.

Clinical observations—including symptoms, signs, biomarkers, and system-level dysfunctions—serve as the entry point for structured inquiry. RIRM organizes mechanistic reasoning through a structured inquiry process, generating outputs that include candidate originating cell(s), mechanistic pathway(s), interacting systems, route classifications, and traceable reasoning steps. These outputs support characterization of underlying mechanisms across cellular, molecular, systemic, adaptive, and emergent system-interaction domains, reflecting the possibility that clinical phenomena may arise from interacting components within complex adaptive biological systems. Mechanistic understanding informs potential pathways toward restoration of functional coherence through normalization, compensation, reorganization, or adaptation. The framework is iterative, with insights from functional outcomes informing the refinement of mechanistic hypotheses over time. For illustrative clarity, only commonly observed routes (A, B, and F) are shown; the complete route classification framework (Routes A–H) is summarized in Table 1. The figure represents a conceptual framework for organizing mechanistic inquiry and should not be interpreted as a validated predictive or analytical model.

**TABLE 1:** Representative illustrative applications of the RIRM across independent projects.

Project	Clinical domain	Investigator-assigned route	Framework-informed route	Mechanistic interpretation	Key insight
006	Neurodegeneration (synaptic dysfunction)	—	A	NMDAR-mediated neuronal signaling with autocrine amplification	High mechanistic coherence consistent with route a reasoning
010	Meibomian gland dysfunction	A	B	PPARγ-driven metabolic signaling without unified remission pathway	Overestimation of mechanistic completeness
020	Carpal tunnel syndrome (TTM)	A	B	Fibroblast-mediated remodeling without molecular convergence	Clinical efficacy insufficiently linked to mechanistic coherence
022	Diabetic neuropathy (TTM)	A	B	Axonal degeneration via sodium channel dysregulation	Incomplete mechanistic cascade
021	Arthrogenic muscle inhibition	F	F	Synoviocyte–muscle–neural axis (inflammatory + reflex)	Correct systems-level classification
029	ALS (brain stimulation)	B	F	Multi-cell neurodegeneration (neurons, glia, immune cells)	Under-recognition of systems complexity
027	Alzheimer’s disease (EEG-based)	B	B	Reduced to synaptic dysfunction	Illustrates reductionist narrowing of mechanistic interpretation
028	Polycythemia vera	B	B	JAK2-driven hematopoietic signaling	Strong signaling pathway without integrated remission framework
025	Brugada syndrome	B	B	Ion channel dysfunction in cardiomyocytes	Valid single-axis model
013	Vascular calcification	—	B	VSMC osteogenic transition (Runx2) with weak receptor linkage	Incomplete mechanistic integration and receptor linkage

Abbreviations: ALS, amyotrophic lateral sclerosis; EEG, electroencephalography; JAK2, Janus kinase 2; NMDAR, N-methyl-D-aspartate receptor; PPARγ, peroxisome proliferator-activated receptor gamma; TTM, Thai traditional massage; VSMC, vascular smooth muscle cell.

Route definitions: Route A, direct mechanistic pathway with defined signaling logic and therapeutic target; Route B, partial mechanistic pathway (e.g., receptor or signaling axis) without complete remission logic; Route C, stage- or progression-based reasoning without mechanistic localization; Route D, cell-centered reasoning emphasizing a dominant originating cell type; Route E, multi-cell interaction without integrated system-level mechanism; Route F, systems-level mechanism involving coordinated multi-cell, multi-tissue, or multi-axis regulation; Route G, adaptive or compensatory physiological mechanism; Route H, unresolved or paradoxical mechanism requiring higher-order integration.

While traditional methodologies advance knowledge through quantitative validation, RIRM begins with qualitative exploration—a disciplined form of inquiry designed to establish conceptual clarity prior to measurement. In this respect, it parallels Grounded Theory in its emphasis on iterative emergence (Glaser and Strauss, 1967; Charmaz, 2014), while extending this logic to molecular and clinical sciences. RIRM re-centers a foundational act of scientific reasoning: the formulation of precise, mechanism-oriented questions about what generates—and restores—functional coherence in disease states (Dewey, 1910; Hmelo-Silver, 2004).

Throughout this perspective, True Remission is used strictly as a conceptual lens rather than a biological endpoint. Within RIRM, it denotes the restoration of functional coherence within a system—whether achieved through normalization, compensation, adaptation, or reorganization. RIRM does not presume a single pathway to health; instead, it provides a framework for organizing inquiry into the diverse and potentially interacting mechanisms through which functional coherence may emerge. Rather than prescribing mechanisms, RIRM aims to make the reasoning processes underlying mechanistic hypothesis generation explicit and open to examination.

## 2 Rationale and conceptual framework

The RIRM was conceived in response to a persistent methodological blind spot in contemporary biomedical research: the absence of systematic frameworks for conceptual discovery. Current research pipelines are optimized for validation rather than generation. Large-scale trials and data-driven analytics refine existing knowledge but rarely guide investigators toward new mechanistic questions (Ioannidis, 2005; Popper, 1959; Lakatos, 1978). Between clinical observation and molecular experimentation lies an under-theorized space—where intuition operates but reproducibility falters (Goodman et al., 2016). RIRM was developed to organize this space and render the underlying reasoning processes more explicit and analytically productive. This gap is particularly evident in conditions in which similar clinical phenotypes emerge from distinct mechanistic pathways across multiple biological scales.

At its core, RIRM treats inquiry itself as a subject of structured analysis. Rather than assuming molecular normalization as the default therapeutic goal, the framework conceptualizes True Remission as the restoration of systemic functional coherence—achieved through normalization, compensation, adaptation, or reorganization of biological processes. This framing is necessary because similar clinical phenomena may arise from fundamentally different mechanistic origins. Anemia, for example, may reflect clonal hematopoietic defects, iron deficiency, endocrine dysfunction, inflammatory signaling, or adaptive physiological states. Likewise, Antarctic icefishes maintain effective oxygen transport despite the evolutionary loss of hemoglobin through alternative physiological adaptations, illustrating that deviation from canonical molecular structures does not inherently constitute pathology (Sidell and O'Brien, 2006). RIRM therefore directs attention toward identifying the mechanistic origins of a clinical problem and reasoning explicitly about how functional coherence is achieved or restored in that context.

Unlike grounded theory, which emphasizes conceptual emergence, or systems biology, which focuses primarily on modeling biological interactions, RIRM aims to make the reasoning processes underlying mechanistic hypothesis generation explicit and open to examination. Its principal contribution lies in providing a route-based framework for organizing mechanistic inquiry and in emphasizing reproducibility of reasoning as a complementary dimension of scientific rigor. Rather than replacing existing qualitative or quantitative traditions, RIRM aims to complement them by making the processes underlying mechanistic discovery more transparent and amenable to critical examination.

The framework organizes this reasoning through two complementary components grounded in inquiry-based learning and qualitative theory-building (Glaser and Strauss, 1967; Charmaz, 2014; Dewey, 1910; Hmelo-Silver, 2004).

### 2.1 Algorithmic roadmap

The algorithmic roadmap comprises eight initial reasoning routes (Routes A-H), each representing a distinct entry logic for mechanistic inquiry, including stage-based, cell-centered, pathogenesis-based, and systems-level perspectives (Supplementary Figure S1). The routes are not hierarchical disease categories, but reasoning archetypes that organize alternative modes of mechanistic inference. Through these routes, researchers may progressively refine the space of plausible explanations toward the most coherent mechanistic interpretation underlying a given clinical presentation. Such interpretation may involve cellular (e.g., clonal hematopoietic disruption), molecular (e.g., defective heme synthesis), systemic (e.g., endocrine dysfunction), adaptive, or emergent mechanisms arising from interacting biological systems.

This process is neither purely deductive nor purely inductive. Rather, it functions as a guided dialectic in which provisional hypotheses are iteratively generated, refined, and examined for biological plausibility (Kuhn, 1962; Lincoln and Guba, 1985). In this respect, the roadmap aligns with systems biology principles emphasizing multi-level integration across cellular, molecular, organismal, and interacting system domains (Kitano, 2002; Noble, 2002; Loscalzo and Barabasi, 2011; Hunter, 2016). Unlike computational models that seek to simulate biological systems, RIRM focuses on the cognitive architecture of mechanistic discovery, making the reasoning processes underlying hypothesis generation explicit and open to examination.

### 2.2 Structured self-inquiry questionnaire

The twelve-item self-inquiry questionnaire serves as the cognitive scaffold for this process (Supplementary Table S1). Each question prompts interrogation of key mechanistic dimensions, including.

- What mechanism—cellular, molecular, systemic, adaptive, or emergent—initiates the clinical phenomenon?
- What constitutes restoration of functional coherence in this context?
- How might this process vary across stages, subtypes, or physiological states, or interacting systems?

By requiring explicit articulation of assumptions and reasoning steps, the questionnaire transforms tacit intuition into structured, reviewable logic (Patel et al., 2005; Croskerry, 2009; Norman and Eva, 2010). Drawing on cognitive science in medicine, it formalizes the reflective cycle of problem representation, hypothesis generation, and iterative refinement (Elstein et al., 2002). In doing so, RIRM renders scientific reasoning both teachable and auditable, establishing a foundation for the reproducibility of reasoning.

To illustrate the conceptual application of the framework, we retrospectively examined representative projects and associated mechanistic reasoning exercises generated during development of the framework. These examples are intended solely as illustrative applications rather than validation exercises and should not be interpreted as human-subject research or evidence of framework performance. Instead, they demonstrate how RIRM may be used to organize mechanistic hypotheses and to reveal inconsistencies, reductionist assumptions, or oversimplifications in mechanistic reasoning across diverse clinical domains. A representative subset is presented in Table 1.

The routes are not hierarchical disease categories, but reasoning archetypes representing different modes of mechanistic inference used during hypothesis generation. Route classification within these illustrative applications was retrospectively determined by investigators involved in development of the framework, based on the degree of mechanistic completeness, system integration, and alignment with principles of functional coherence. Across projects, investigators frequently favored single-axis explanations (Route A/B), even in conditions characterized by established multi-cell or multi-system pathophysiology. These observations suggest that reductionist tendencies may commonly arise in mechanistic reasoning and illustrate the types of patterns that RIRM is intended to make explicit.

#### 2.2.1 Integration through a digital reasoning architecture

The two components of the framework—the algorithmic roadmap and the structured self-inquiry questionnaire—are conceptually compatible with a digital reasoning architecture that treats scientific inquiry as an analyzable process. Within such an architecture, reasoning may be represented as a sequence of structured decisions, assumptions, and inferential transitions rather than remaining implicit or intuitive.

This representation enables traceability of reasoning pathways, including the selection of mechanistic entry points, the generation and rejection of hypotheses, and the articulation of criteria for functional coherence. Such traceability aligns with broader movements in data-centric science that emphasize transparency not only of results but also of the reasoning processes that generate them (Leonelli, 2015). In early-stage biomedical research—where mechanistic hypothesis generation is often guided by tacit expertise—making reasoning explicit may facilitate critical evaluation, comparison, and reuse.

Importantly, this digital dimension is presented as a conceptual extension rather than an implemented system. Its purpose is to illustrate how structured inquiry might support the reproducibility of reasoning—an underdeveloped dimension of scientific rigor that complements, rather than replaces, quantitative validation (Goodman et al., 2016). No software implementation or computational framework is proposed or evaluated in the present work.

In this sense, RIRM situates mechanistic hypothesis generation within a systems-oriented epistemology in which reasoning processes can be represented, examined, and iteratively refined across multiple levels of biological interpretation.

#### 2.2.2 Epistemic positioning

Although RIRM allows reasoning to be represented in structured forms, its epistemic foundation remains qualitative. The framework is not intended to test statistical differences or generate empirical claims, but rather to formalize the reasoning processes that precede quantitative experimentation (Glaser and Strauss, 1967; Charmaz, 2014). It is at this pre-experimental stage that many mechanistic hypotheses emerge, yet the underlying reasoning is often left tacit, intuitive, and difficult to reproduce.

By making this phase explicit, RIRM occupies a conceptual space between two scientific traditions: the interpretative depth of grounded theory and the structural discipline of systems medicine. Within this synthesis, rigor extends beyond measurable outcomes to include the clarity, coherence, and transparency of reasoning itself. Scientific inquiry is thus reframed as an epistemic process—open to scrutiny, comparison, and iterative refinement across multiple levels of interpretation.

Importantly, RIRM is intended as a conceptual framework for organizing mechanistic inquiry rather than as a validated methodology, predictive model, or analytical system. Its purpose is to make the processes underlying mechanistic hypothesis generation more explicit and open to examination, thereby complementing rather than replacing empirical investigation.

## 3 Discussion and future directions

RIRM challenges conventional boundaries in biomedical research by treating inquiry itself as a process open to structured analysis. While modern science has optimized the measurement of outcomes, it has not achieved comparable rigor in the reproducibility of reasoning (Goodman et al., 2016). Experimental reproducibility itself may be limited when the intellectual steps leading to an experiment remain undocumented or implicit (Ioannidis, 2005). RIRM addresses this gap by proposing that the structure of reasoning—the sequence of questions, assumptions, and inferential transitions guiding mechanistic inquiry—should be made explicit, teachable, and open to evaluation (Popper, 1959; Lakatos, 1978). This study does not present empirical validation, and the illustrative applications are intended to demonstrate conceptual utility rather than clinical effectiveness, predictive performance, or reproducibility of route assignment.

This orientation aligns with broader movements in the philosophy of science emphasizing epistemic transparency: understanding how knowledge is generated, not only what is known (Kuhn, 1962; Leonelli, 2015). By conceptualizing reasoning as a structured and iterative process, RIRM elevates early-stage hypothesis generation from an informal activity to a legitimate domain of scientific rigor.

RIRM also expands the systems-biology conception of mechanism. Rather than assuming that health corresponds to correction of a single structural or molecular deviation, the framework accommodates mechanisms that are cellular, molecular, systemic, adaptive, or emergent through interacting biological systems. Restoration of functional coherence may involve normalization, compensation, reorganization, or adaptive system responses, reflecting the diversity of biological solutions observed across disease states and evolutionary contexts.

Future work should prospectively evaluate the reproducibility of route assignments and the degree of agreement among independent investigators. Such studies may help determine whether structured mechanistic inquiry can improve transparency, consistency, and reproducibility of reasoning across biomedical domains. These questions lie beyond the scope of the present Perspective but represent important directions for further development and empirical evaluation of the framework.

### 3.1 Bridging qualitative and quantitative thinking

RIRM occupies a conceptual space between qualitative inquiry and systems-level analysis. From grounded theory, it adopts iterative sense-making and conceptual emergence (Glaser and Strauss, 1967; Charmaz, 2014). From quantitative scientific traditions, it adopts structural discipline, explicit inferential pathways, and reproducibility-oriented reasoning (Goodman et al., 2016; Popper, 1959). Together, these elements form a hybrid epistemology—structured qualitative reasoning—that preserves interpretive depth while enabling systematic comparison, refinement, and mechanistic integration across multiple levels of analysis.

Unlike grounded theory, which primarily emphasizes conceptual emergence, or quantitative approaches that prioritize empirical validation, RIRM focuses on making the reasoning processes underlying mechanistic hypothesis generation explicit and open to examination. In this respect, it aims to complement rather than replace existing qualitative and quantitative traditions.

Importantly, RIRM does not replace quantitative validation. Rather, it strengthens the reasoning processes that precede measurement, improving the coherence and mechanistic quality of hypotheses entering empirical testing. In this sense, rigor is reframed not only as a property of results, but also of the reasoning that generates them.

### 3.2 Transformative potential

As a conceptual framework, RIRM offers a structured approach for generating, organizing, and articulating mechanistic hypotheses. For researchers, it provides a transparent approach for specifying assumptions and inferential pathways prior to experimentation. For educators, it offers a scaffold for teaching clinical reasoning and pathophysiology as processes of structured inquiry (Norman and Eva, 2010; Elstein et al., 2002). For interdisciplinary teams, it provides a shared conceptual language for integrating molecular, clinical, systems-level, and adaptive perspectives (Noble, 2002; Hunter, 2016).

More broadly, RIRM points toward the possibility of cognitive analytics in medicine—the systematic study of how scientific reasoning unfolds in practice, not only what discoveries result (Leonelli, 2015). Such an approach may support future integration between human reasoning and computational systems without reducing inquiry to algorithmic substitution. Instead, computational tools may augment transparency, comparison, and refinement of mechanistic reasoning across multiple biological and conceptual scales.

## 4 Conclusion

RIRM is a conceptual framework rather than an empirically validated methodology. Its principal contribution lies in formalizing inquiry itself as a legitimate domain of scientific attention by providing an epistemic framework for organizing mechanistic reasoning in biomedical research. By structuring the process of questioning, RIRM reframes remission research as a disciplined search for the mechanisms through which functional coherence is achieved, whether through normalization, compensation, adaptation, reorganization, or emergent system interactions within complex biological systems.

More broadly, the framework encourages a shift from focusing exclusively on outcomes toward examining the reasoning processes that generate them. Scientific progress depends not only on better data, but also on more transparent, structured, and reproducible approaches to mechanistic inquiry. By making reasoning processes explicit, comparable, and open to refinement, RIRM seeks to strengthen the conceptual foundations of biomedical discovery across multiple levels of biological interpretation. Future empirical studies will be required to evaluate the reproducibility, consistency, and practical utility of the framework across independent investigators and biomedical domains.

## Data Availability

The original contributions presented in the study are included in the article/Supplementary Material, further inquiries can be directed to the corresponding author.
