# Supplementary material for: The Remission Therapy Inquiry-Based Research Model (RIRM): a conceptual framework for mechanistic hypothesis generation
Source: Front Syst Biol. 2026 Jul 8;6:1835804. doi: 10.3389/fsysb.2026.1835804 (PMC13389765; doi:10.3389/fsysb.2026.1835804)
Supplement: Supplementary file 1 [file Supplementaryfile1.docx]

**Supplementary Figure S1. Complete route framework of the Remission Therapy Inquiry-Based Research Model (RIRM).**


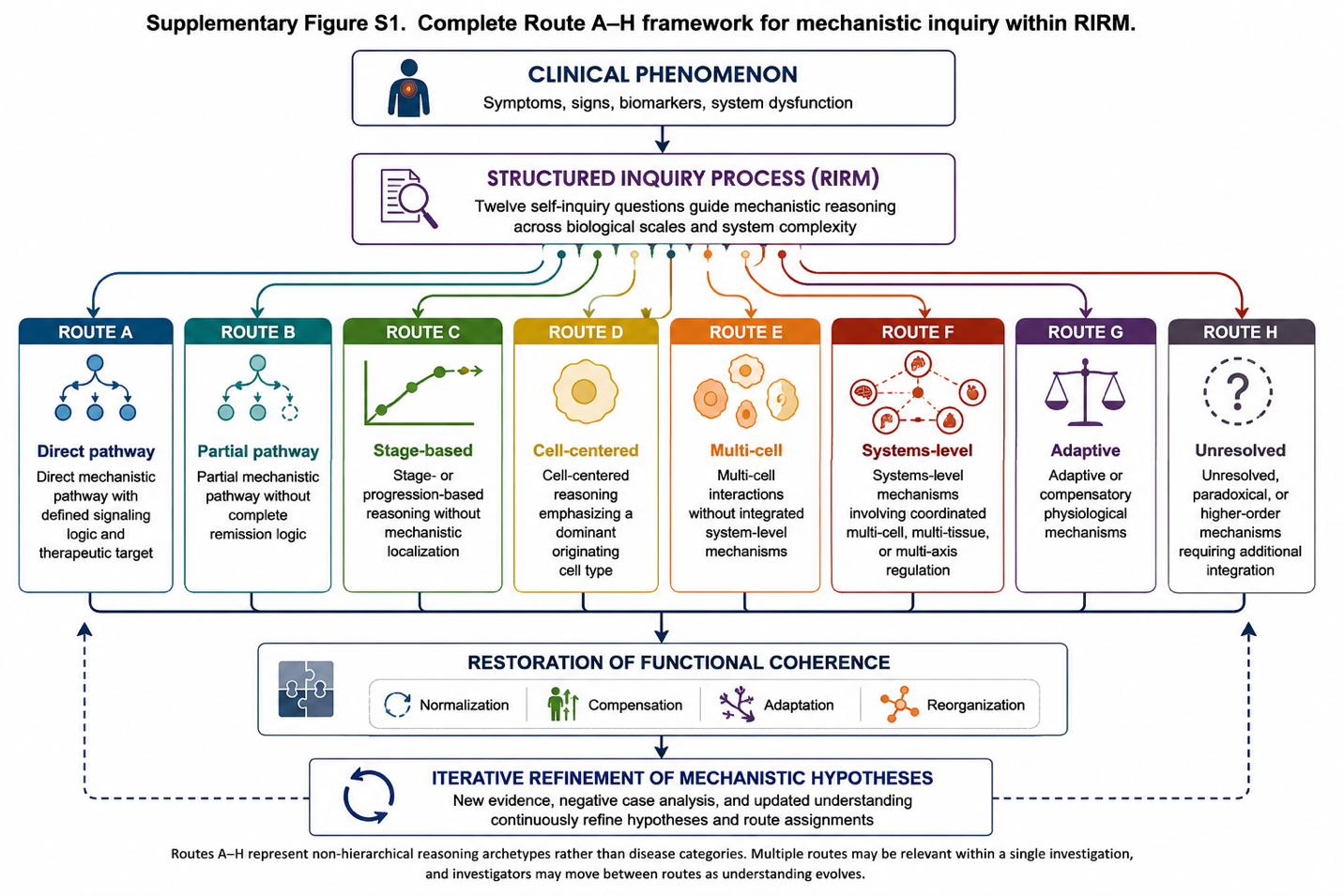


Following clinical observation and structured inquiry, investigators may enter mechanistic reasoning through eight non-hierarchical routes (Routes A–H), each representing a distinct reasoning archetype rather than a disease category. These routes encompass direct molecular pathways, cell-centered mechanisms, multi-cell interactions, systems-level regulation, adaptive responses, and unresolved mechanisms. Mechanistic interpretations inform pathways toward restoration of functional coherence and are iteratively refined as new evidence emerges. The framework is intended as a conceptual scaffold for organizing mechanistic inquiry and should not be interpreted as a validated predictive or analytical model.
