# Supplementary material for: The Remission Therapy Inquiry-Based Research Model (RIRM): a conceptual framework for mechanistic hypothesis generation
Source: Front Syst Biol. 2026 Jul 8;6:1835804. doi: 10.3389/fsysb.2026.1835804 (PMC13389765; doi:10.3389/fsysb.2026.1835804)
Supplement: Supplementary file 2 [file Table1.docx]

**Supplementary Table S1. Twelve self-inquiry questions comprising the Remission Therapy Inquiry-Based Research Model (RIRM)**

| **Domain** | **Self-inquiry question** |
| --- | --- |
| Clinical phenomenon | 1. What clinical phenomenon requires explanation? |
| Origin | 2. Which cell, tissue, organ, or system is most likely to initiate the observed dysfunction? |
| Mechanism | 3. What mechanism—cellular, molecular, systemic, adaptive, or emergent—could explain the phenomenon? |
| Pathway | 4. Which signaling pathways, physiological processes, or interacting systems are involved? |
| Scale | 5. At what biological level(s) does the dysfunction primarily occur? |
| Heterogeneity | 6. Could different stages, subtypes, or contexts involve distinct mechanisms? |
| Interactions | 7. Are multiple cells, tissues, or systems interacting to produce the phenotype? |
| Adaptive responses | 8. Could compensatory or adaptive mechanisms contribute to the observed state? |
| Functional coherence | 9. What constitutes restoration of functional coherence in this context? |
| Therapeutic logic | 10. Through what mechanisms might restoration be achieved (normalization, compensation, adaptation, or reorganization)? |
| Alternative explanations | 11. What competing mechanistic hypotheses should be considered? |
| Iterative refinement | 12. How should new evidence modify or refine the current mechanistic interpretation? |
